# Development and Pilot Implementation of a Training Framework to Prepare and Integrate Pharmacy Students into a Multicentre Hospital Research Study

**DOI:** 10.3390/pharmacy10030057

**Published:** 2022-05-30

**Authors:** Aaron Noble, Rachael Raleigh, Amy Page, H. Laetitia Hattingh

**Affiliations:** 1 Pharmacy Department, Gold Coast Health, Gold Coast, QLD 4215, Australia; aaron.noble@health.qld.gov.au (A.N.); rachael.raleigh@health.qld.gov.au (R.R.); 2 Pharmacy Department, Alfred Health, Melbourne, VIC 3000, Australia; amy.page@monash.edu; 3 School of Pharmacy and Medical Science, Griffith University, Gold Coast, QLD 4222, Australia

**Keywords:** pharmacy student, research participation, medication related harm, hospital pharmacy research

## Abstract

A unique approach was introduced to integrate pharmacy students into a multicentre patient-centred research project predicting medication related harm (MRH) post-discharge. A training framework was developed to prepare students for research participation and integration. The framework aligned research project tasks with the pharmacists’ national competency standards framework. The framework was piloted on four research placement students from two local universities during three hospital placements, from October 2020 to August 2021. Following their initial orientation and training, students collected data from 38 patients and were involved in patient screening processes, interviewing, data collection and analysis. Patients’ MRH risk scores correlated with re-admission rates with 16/38 (42%) of patients re-admitted within eight weeks following discharge. Their participation in the research enabled students to obtain skills in (1) literature searching, (2) maintaining patient confidentiality, (3) interviewing patients, (4) obtaining data from medical records, (5) communicating with patients and clinicians, and (6) the use of clinical information to predict MRH risk.

## 1. Introduction

Pharmacy students are required to participate in research as part of their degree programs to develop a basic level of research skills [1]. In addition to the development of research skills, studies have shown that the integration of research into degree programs resulted in improved student satisfaction [2,3]. Research exposure was perceived as beneficial by students [4], facilitated their evolvement as practitioners and innovators [5], improved their perceptions of the usefulness and importance of research [6], improved their ability to work/think independently [7] and developed positive attitudes towards research [8]. Mentors/preceptors also viewed research as a positive and valuable learning experience for the students [9,10]. Involving pharmacy students in research that focuses on medicine information transfer during transitions of care provides unique insights and skills in processes to minimise medication related harm (MRH).

MRH is a major public health issue in Australia estimated to cause 400,000 emergency department and 250,000 hospital admissions annually with an estimated annual cost of AUD 1.4 billion [11,12]. In the United Kingdom (UK), MRH in the eight weeks following discharge costs the government GBP 400 million annually [13]. A patient risk analysis tool, the PRIME tool (Prospective study to develop a model to stratify the Risk of Medication related harm in hospitalised Elderly patients), was developed in the UK to identify elderly patients at high risk of MRH following hospital discharge [14]. A need was identified to validate the PRIME tool in other countries. An Australian multisite MRH research project, led by the Alfred Hospital in Melbourne, Victoria, was established with the Gold Coast Hospital and Health Service (GCHHS, Queensland, Australia) as one of the study sites.

This study focused on the unique processes developed and followed by mentor pharmacy students during research placements when participating in project data collection. The participation of students in a multicentre research project was considered unique as pharmacy placement student projects are traditionally audit-type quality use of medicine projects at GCHHS. Involvement in the MRH research project provided an opportunity for students to gain experience of and exposure to a bigger research project, including having interactions with consenting patients for data collection. There was, however, a need to develop a training framework to prepare students for their participation in the project. This paper describes the processes followed during the development and pilot implementation of a research training framework for students to develop research, clinical and communication skills.

## 2. Materials and Methods

The multisite project received Human Research Ethics Committee approvals from Alfred Health and the GCHHS HREC/59539/Alfred-2019-196445 on 30 March 2020. The prospective cohort study followed the STROBE checklist [15].

The development and implementation of the research training framework was guided by the Medical Research Council’s guidelines for complex interventions, which outlines a systematic process for developing interventions based on evidence and concepts/theory [16]. The guidelines recommend piloting a research design before evaluation of intervention effectiveness and implementation into practice. There are four steps: (1) intervention development, (2) piloting, (3) evaluation and (4) implementation [16]. This study focused on steps 1 and 2 to allow gathering of useful insights of how the framework was applied for future replication, evaluation and implementation. Specific objectives were to:

1. Develop a research training framework to facilitate the participation of pharmacy students in hospital pharmacy patient-centred research.
2. Pilot the training framework through the integration of pharmacy students in the MRH research project.

This pilot study focused on processes to inform future implementation [17,18] and followed the CONSORT framework [19].

### 2.1. Setting

This study was conducted at GCHHS that incorporates two public hospitals with over 1350 funded beds. Gold Coast University Hospital is one of Queensland’s largest clinical teaching and research facilities, whilst Robina Hospital is part of a large health hub with adjacent health services and health precincts [20]. Participating pharmacy students collected data from consenting patients at both hospitals.

### 2.2. Development of Research Training Framework

The MRH research project stages were mapped against the 2016 National Competency Standards Framework (NCSF) for Pharmacists in Australia. This process was followed to develop a training framework that would align the identified competency domains with the research project to guide students’ learning process. Four of the five domains from the NCSF were identified as relevant [21]:

- Professionalism and ethics,
- Communication and collaboration,
- Medicines management and patient care, and
- Education and research.

### 2.3. Pilot Implementation

The implementation of the training framework was piloted between October 2020 and August 2021. Final year pharmacy students were recruited from two local universities over three placement cycles. A placement cycle is a block of 3–4 weeks of a full-time clinical hospital placement. Due to the length of a cycle, we aimed to have the students participate in specific components of the research journey as it was not practical to have them participate from project conception to publication given the timeframe. The project was presented to Bachelor of Pharmacy (BPharm) and Master of Pharmacy (MPharm) students as an option for their clinical placements. Students from the one university had to apply to be allocated to the project through a competitive process before the start of the placements. Students from the other university could request allocation to the project at the commencement of their placements. The recruitment process was therefore a mix between a competitive process and opting to participate.

Selected students were briefed on the site-specific project standard operating procedure (SOP) and the roles and responsibilities of the various team members. The SOP provided background information, the overall study process and guidance for students regarding which team member to contact if they had any questions. For issues concerning the study process, students were directed to contact a research pharmacist, whilst for clinical patient issues, students were to contact the inpatient unit pharmacist (IPU) looking after participating patients.

An in-house clinical prioritisation tool was used to screen potential patients at high risk of MRH considering research project inclusion criteria: over 65 years of age, anticipated discharge from a general medical IPU within 48 h and taking more than one regular medication. Exclusion criteria were non-English speaking, lack of capacity, cognitive impairment or transferring to an acute or sub-acute medical facility. Potential participants were provided the participant information and consent and study withdrawal forms and offered sufficient time to consider involvement in the study. Participants provided written consent and contact details for follow-up interviews.

### 2.4. Data Collection

As this is a descriptive study focused on processes followed, no hypothesis was tested and therefore no formal sample size calculation was undertaken. However, in accordance with the literature on sample sizes for pilot and feasibility studies, the aim was for the students to recruit at least 35 patients [22]. Various data points provided information on patient participants’ potential risk of MRH:

- Patient interviews prior to discharge: Face-to-face interviews were conducted within the 48-h period before discharge from hospital. Interview questions followed an interview guide, designed to validate the PRIME tool. The interview guide incorporated validated tools to capture data on participants’ social situation, mental health, cognition, medication management and activities of daily living.
- Data from the electronic medical records that included participant observations, pathology results and medication regime on both admission and discharge.
- Patient telephone interviews at two weeks, four weeks and eight weeks post the discharge date. Interviews incorporated questions on how the participant had been since discharge, any adverse effects experienced and questions using the MUSE scale (medication understanding and use self-efficacy scale). The utilisation of healthcare was checked, and for each encounter, the date and reason documented.
- Discharge pharmacist interviews: A structured interview guide was used to explore pharmacists’ opinions on how likely the patient might be re-admitted due to MRH and their confidence in their responses. The final questions included an estimation from the pharmacist on the likelihood of the patient accessing community healthcare in the following eight weeks due to MRH and whether they counselled the patient on their medication on discharge.

Student progress was monitored daily through regular meetings with research team members, student diaries and field notes, and preceptor notes and records. Students were invited to provide email feedback at the end of their placements.

### 2.5. Data Analysis

Baseline data were used to calculate each participant’s potential MRH risk through application of the PRIME tool formula. The baseline data in REDCap© was checked to verify patient interview data against the electronic medical records and to explore the records of those patients who were re-admitted to hospital. Detailed information was recorded in MS Excel© to compare MRH risk against re-admitted episodes.

## 3. Results

Four students participated in the research project over three cycles: cycles 1 and 2 (October 2020; February to March 2021) focused on patient selection, consenting, baseline data collection and the interviewing of patients and pharmacists, whereas the 3rd cycle (July 2021) focused on patients’ MRH risk calculation. The 1st cycle involved two MPharm students over a 3-week period and the 2nd and 3rd cycles one BPharm student each over 4-week periods.

### 3.1. Development of Research Training Framework

The framework to guide the separation of roles and responsibilities between students and research pharmacists is summarised in Table 1 with details on students’ research involvement.

### 3.2. Implementation and Evaluation of the Training Framework

Students were briefed on the site-specific standard operating procedure (SOP) and the roles and responsibilities of the various team members. The SOP provided background information, the overall study process and guidance for students regarding which team member to contact if they had any questions. All study steps were explained to all students with practical examples of how to complete the various tasks. The cycle 1 and 2 students conducted role plays to practise interview skills, whilst the cycle 3 student was shown how to extract data and calculate potential MRH risk. The four students successfully collected data from 38 consenting patients and were involved in patient screening processes, the interviewing of patients and pharmacists, data collection and analysis, the follow-up patient interviews and MRH risk calculations.

Sixteen of the participants (42%) were re-admitted to hospital within the 8-week follow-up post-discharge, which included a total of 27 hospital encounters. The patient group who had representations/re-admissions to hospital had a higher average MRH risk score than the participants who did not have any post-discharge encounters (36.7% v 29.7%), using the PRIME tool formula [14]:

PRIME Risk score = −2.384 + 0.85 × 0.025 (age, 81)) − 0.398 (* gender) + 0.515 (* antiplatelet medicine) − 0.042 (sodium-137) + 0.591 (* antidiabetic medicine) + 0.477 (* past adverse drug reaction, ADR) + 0.056 (number of medicines) + 0.397 (* living alone).

* Gender (female = 0), antiplatelet medicine (antiplatelet on discharge = 1), antidiabetic medicine (antidiabetic on discharge = 1), past ADR (past ADR = 1) and living alone (living alone post-discharge = 1). Individual estimated risk of MRH (%) = (1/1 + e^−risk score^) * 100.

Patients who were re-admitted to hospital had higher average risk scores for the following PRIME risk factors compared to those who had not been re-admitted:

- Age
- Gender
- Number of medicines
- Sodium level (mmol/L)
- Antiplatelet medicine

Table 2 outlines the skills the pharmacy students were exposed to, developed and built on as the study progressed, measured against the NCSF [21]. Participation in the research project enabled students to address and obtain skills in the four identified competency domains, specifically incorporating skills in maintaining patient privacy and confidentiality, literature searching and the critical evaluation of resources, interviewing patients, obtaining data from medical records, communicating with patients and clinicians and use of clinical information to predict patients’ potential MRH risk.

### 3.3. Student Feedback

Students’ feedback about the research placement and the skills learnt was very positive. The placement provided an opportunity for students to learn about the role of hospital pharmacists in discharge medicine handover and continuity of care:

*“The exposure this placement gave me to hospital pharmacy, the skills I developed and opportunities it gave me greatly benefited my learning as a student and provided me with foundational skills and knowledge … Under supervision, I gained an understanding of the roles and responsibilities of a ward pharmacist and the multidisciplinary environment of the hospital. Through this placement I gained a passion for the role pharmacy has in facilitating medication management to patients and healthcare staff and the importance of continuity of care post discharge”.*

Importantly, the placement also exposed students to hospital pharmacy research and the value of research in practice:

*“This placement was the first time I understood the importance of research within pharmacy and the beneficial application it can have to current practice. … I loved this placement and am grateful for the opportunity I had to learn from supervising registered pharmacists, meet patients and experience hospital pharmacy which has benefited me greatly as I progress as a learning pharmacist. Most of all I am glad I had the opportunity to see the importance of using research to address gaps in practice so that we as pharmacists are providing the best care to patients and upholding our responsibility to ensure the safe and effective use of medicines.”*

## 4. Discussion

The pilot implementation of the research training framework showed it provided a useful structure to prepare pharmacy students for active involvement in a hospital patient-centred research project. Participation in the research project enabled students to obtain skills in a range of competency domains from the NCSF, namely professionalism and ethics, communication and collaboration, medicines management and patient care, and education and research. The training framework facilitated student participation in research whilst also developing and building essential skills applicable to pharmacy practice.

The American Society of Health-System Pharmacists recently defined steps to involve pharmacy students in research [23]. Our framework followed a similar approach and provided a useful structure to prepare pharmacy students for active involvement in a hospital patient-centred research project. Previous studies highlighted the need for the proper preparation of pharmacy students before exposing them to research [24,25]. A Malaysian study that surveyed 128 final year pharmacy students reported that 91.4% felt they were under stress whilst conducting research and that additional support and preparation improved students’ self-efficacy and reduced research anxiety [26]. An Australian survey of pharmacy students across each year of the 4-year degree program similarly showed that almost half of the 853 respondents lacked confidence to undertake research despite most agreeing that research played an important part in the profession [27]. An American study that focused on pharmacy students’ barriers and facilitators that influence research participation showed that 81.8% of 623 surveyed students felt unfamiliarity with the research process was a main barrier to undertaking research. Of interest is that the study also showed that students who were engaged in research during their studies were interested in clinical research and were more likely to pursue postgraduate training opportunities [28]. Our training framework prepared students for their involvement in the research project and facilitated the provision of ongoing support throughout the research placements. Student feedback was very positive and showed that the training framework fostered a positive learning environment and an appreciation for the value of hospital pharmacy research.

The importance of incorporating research and scholarly activity into pharmacy training was recognised by the American College of Clinical Pharmacy in 2016 [29]. One of the suggestions made by the College was to enhance research involvement through field experiences. Our study provided such an opportunity through the involvement of pharmacy students in a multicentre research project during their hospital placements. The College also highlighted the importance of early engagement in research activities in contributing to professional growth, the development of critical thinking and time management skills, and engendering a cognitive approach to solving health care challenges [29]. Our training framework indeed exposed students to a range of competency areas including an understanding of professionalism and ethics. Studies have shown that participation in research can help to foster the development of clinical reasoning skills and emphasise the importance of evidence-based practice [6]. These skills are not only relevant skills in hospital pharmacy practice but also in community pharmacy practice to address MRH during the transition between hospital and primary care. This transition period is considered to be the highest risk period for MRH [30,31,32,33].

The development of a research rubric to define core research competencies implemented by university faculty members and incorporated into university assessments showed positive results in gaining research skills [34]. A study conducted in California, United States of America, that used peer training through a model where senior students trained junior students under the supervision of a research mentor showed increased research outputs [35]. Our study similarly showed positive outcomes in the gaining of research skills. It was unique in that we developed a research training framework to engage pharmacy students in research in a hospital setting. Placement outcomes showed that the development of the training framework facilitated the identification of required student skills and competencies against the NCSF competency domains [21]. The training framework allowed the placement students to be exposed to and gain experience in research whilst also developing and building essential skills applicable to pharmacy practice.

Almost half of the study participants (42%) were re-admitted within eight weeks of discharge from hospital with a total of 27 hospital encounters. Further analysis is needed to determine the potential causes and whether these re-admissions were linked to MRH post-discharge. Pharmacists can play a key role in the prevention of potential MRH on discharge by ensuring communication in medication changes are clearly documented and communicated to the patient/carer/family or a significant stakeholder. The World Health Organisation in 2017 identified MRH as a priority global patient safety challenge with the aim to reduce severe avoidable medication related harm by 50% over the next five years [36]. Being the medicine experts, pharmacists play a key role in identifying and implementing solutions to avoid potential MRH, acknowledging that most MRH arises from the failure of systems involved in patient health care due to the number of potential medical providers involved in each patient care [37,38,39,40]. Exposure of the pharmacy students to the MRH research project and specific research tasks facilitated the development of unique pharmacy practice skills to address MRH.

The strength of the study lies in the structured processes followed to develop the framework, including the integration of the NCSF competency domains. This prompted the following of robust processes and streamlining of the training according to individual student’s baseline skills. A limitation was the small number of participating students: the application of the framework was only piloted on four students as placements were impacted by the COVID-19 pandemic. Due to the COVID-19 pandemic, hospital requirements changed during 2021 and pharmacy students were not allowed to have contact with patients. As students are offered placements at several institutions in the local area who all provide multiple project opportunities, the number of students on offer to any project is limited. It is possible that there could have been selection bias by the research team in the selection of the students who showed an interest in being a part of the project.

## 5. Conclusions

The development of a student research training framework provided a platform to define pharmacy students’ training needs prior to their involvement in a hospital research project. This approach enabled the research team to prepare and mentor students for patient-centred hospital research. The involvement of the pharmacy students facilitated research in a hospital pharmacy department with limited resources devoted to research. The training framework developed will be refined and evaluated on an ongoing basis and could be adapted for other research projects to develop students’ research skills and facilitate hospital pharmacists’ participation in research.

## Figures and Tables

**Table 1:** Allocation of roles between pharmacy students and research pharmacists within the research training framework.

Study Stage	Research Pharmacist Roles	Pharmacy Student Roles	Tasks Completed and Skills Obtained by Pharmacy Student as Part of the Research Training Framework
Student preparation	Discussed standard operating procedure (SOP) and role of team members Organised access to hospital integrated electronic Medical Record (ieMR) system and online research database (REDcap ©) Demonstrated how to find electronic information and data entry Discussed relevant literature	Familiarised with the SOP Received access to hospital electronic medical record system and online research database Literature review	Completed training activities prior to data collection. This included: - ieMR data collection practice - Data input into the online research database REDCap © - Interview training and preparation - Literature review of at least five related peer reviewed journal articles
Patient selection	Identified potential patients by applying inclusion and exclusion criteria	Observed pharmacist	Understood rationale for inclusion and exclusion criteria Obtained any additional relevant details from inpatient unit (IPU) pharmacist not documented in medical records
Participant consent	Explained participant information and consent form with participant	Observed pharmacist	Observed research pharmacists consenting eligible participants Entered patient details into master Excel © spreadsheet to document follow-up interview timeframes
Baseline participant interview	Conducted interview with student observing. Observed student interviewing and when competent, allowed student to interview without direct supervision	Initially observed, then interviewed participant under supervision until competent to interview independently	Participated in interview role play and observation of research pharmacist conducting baseline interview Conducted baseline interview under supervision until considered competent Completed baseline interview without supervision If issues arose during interview, ceased interview and followed appropriate escalation step as per SOP
Baseline medical data collection	Checked accuracy of data obtained and entered into research database	Obtained data from medical records and entered into research database	Collected and entered medical data from ieMR into Redcap^©^
Discharge pharmacist interview	Demonstrated interview as student observed. Observed student then allowed student to interview without direct supervision	Initially observed, then interviewed with pharmacist supervision. Once competent, interviewed independently	Conducted discharge interview at convenient time with discharging IPU pharmacist
Week 2 follow-up patient interview	Conducted patient telephone interview that fell outside student placement timeframe	Student conducted patient telephone interviews (if fell within placement timeframe)	Conducted 2-week follow-up interview via phone Entered data and impression of participant adherence into REDCap ©
Week 4 follow-up patient interview	Conducted patient telephone interviews	Fell outside placement timeframe	NA
Week 8 follow-up patient interview	Conducted patient telephone interviews	Fell outside placement timeframe	NA
Medication related harm (MRH) risk calculation	Supervised calculation of MRH	Checked medical records and follow-up interview data Calculated re-admitted patients’ risk of MRH post-discharge Compared calculated risk with re-admission history	Obtained applicable data from baseline data and medical records to calculate potential MRH risk using the PRIME tool formula

ieMR: integrated electronic Medical Record; IPU: inpatient unit; MRH: medication related harm; standard operating procedure (SOP).

**Table 2:** Skills developed by the students mapped against the National Competency Standards Framework for Pharmacists [21].

Domain	Standard	Competency	Skill Developed by Student	Stage Skill Developed					
				SP	PS	BI	BDC	FUI	DA
One: Professional and ethics	Practice within applicable legal framework	Respect and protect individual’s right to privacy and confidentiality	Apply patient confidentiality with each participant having their own unique identifier in the research database	✓					
		Assist individuals to understand and grant informed consent	Exposed to study consenting processes		✓	✓			
	Contribute to continuous improvement in quality and safety	Collaborate to improve quality and safety across the continuum of care	Develop skills in application of clinical information to identify patients at risk of medication related harm	✓	✓		✓		✓
Two: Communication and collaboration	Communicate Effectively	Use appropriate communication skills	Develop essential communication and counselling skills to obtain medical data and communicate effectively with each participant - Develop rapport - Learn to minimise jargon - Develop, recognise and use verbal/non-verbal cues			✓		✓	
Three: Medicines management and patient care	Develop a patient-centred, culturally responsive approach to medicine management	Obtain relevant health and medicines information	Experience and practise obtaining medical data directly from patients during the baseline interview or from the electronic medical database			✓	✓	✓	✓
Five: Education and research	Participate in research	Undertake critical evaluation activities	Conducted an initial literature review to evaluate literature sources and provide five related references to the study		✓	✓	✓	✓	✓
	Research, synthesise and integrate evidence into practice	Retrieve relevant information/evidence in a timely manner	Developed time management skills to ensure the medical information was obtained without delaying discharge processes and facilitate contacting of patients within the required timeframe			✓	✓	✓	✓

SP: Student Preparation; PS: Patient Selection; BI: Baseline Interview; BDC: Baseline Data Collection; FUI: Follow-up Interview, DA: Data analysis.

## Data Availability

Not applicable.
